# Invasion trajectory of Pacific oysters in the northern Wadden Sea

**DOI:** 10.1007/s00227-017-3104-2

**Published:** 2017-03-06

**Authors:** Karsten Reise, Christian Buschbaum, Heike Büttger, Johannes Rick, K. Mathias Wegner

**Affiliations:** 1 Alfred Wegener Institute, Helmholtz Centre for Polar and Marine Research, Wadden Sea Station Sylt, Hafenstr. 43, 25992 List, Bremerhaven, Germany; 2 BioConsult SH, Schobüller Str. 36, 25813 Husum, Germany

## Abstract

Invasion trajectories of introduced alien species usually begin with a long establishment phase of low abundance, often followed by exponential expansion and subsequent adjustment phases. We review the first 26 years of feral Pacific oysters *Crassostrea gigas* around the island of Sylt in the Wadden Sea (North Sea, NE Atlantic), and reveal causal conditions for the invasion phases. Sea-based oyster farming with repeated introductions made establishment of feral oysters almost inevitable. Beds of mussels *Mytilus edulis* on mud flats offered firm substrate for attachment and ideal growth conditions around low tide level. *C. gigas* mapped on to the spatial pattern of mussel beds. During the 1990s, cold summers often hampered recruitment and abundances remained low but oyster longevity secured persistence. Since the 2000s, summers were often warmer and recruitment more regular. Young oysters attached to adult oysters and abundances of >1000 m^−2^ were achieved. However, peak abundance was followed by recruitment failure. The population declined and then was also struck by ice winters causing high mortality. Recovery was fast (>2000 m^−2^) but then recruitment failed again. We expect adjustment phase will proceed with mean abundance of about 1000 m^−2^ but density-dependent (e.g., diseases) and density-independent (e.g., weather anomalies) events causing strong fluctuations. With continued global warming, feral *C. gigas* at the current invasion fronts in British estuaries and Scandinavian fjords may show similar adjustment trajectories as observed in the northern Wadden Sea, and also other marine introductions may follow the invasion trajectory of Pacific oysters.

## Introduction

Transfers of marine organisms in the wake of expanding global markets and seafaring are increasingly changing the species compositions along coasts (Rilov and Crooks 2009). Whether nonnative species become established at a distant coast depends on frequency and magnitude of introductions (propagule pressure *sensu* Johnston et al. 2009), on invasive traits, on physiological matches with and adaptations to environmental conditions, and on the invasibility of recipient ecosystems (Olyarnik et al. 2009). Invasions often proceed through a prolonged lag phase before exponential population growth commences (Crooks 2005; Crooks and Rilov 2009). Eventually growth will stop and population abundance decline again (Simberloff and Gibbons 2004). How prevalent such patterns are in marine ecosystems will require more detailed long-term studies on invasions stemming from various organismal groups (Lockwood and Robinson 2014).

**Fig. 1:**
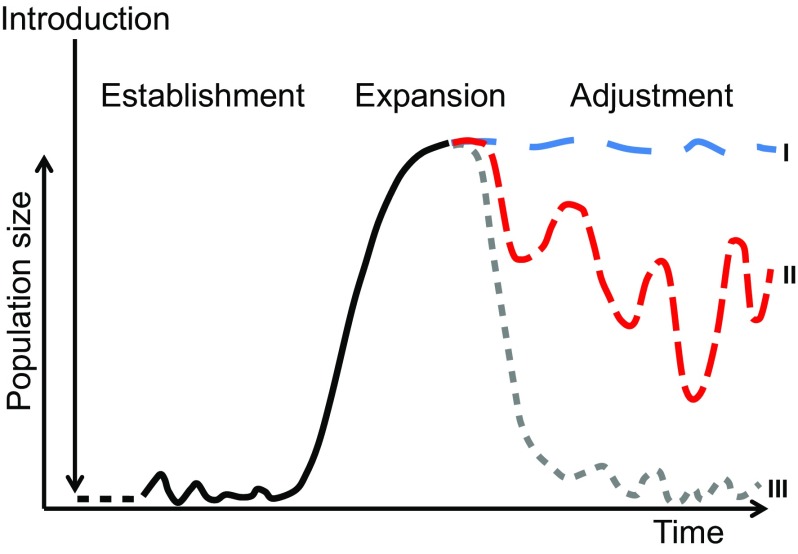
Invasion trajectories after one or more introductions with consecutive establishment, expansion and adjustment phase. Dominance may be maintained during the latter phase (*type I*), strong fluctuations may be caused by environmental, intrinsic and enemy effects (*type II*) or rarity prevails after boom and bust (*type III*)

To compare invasion trajectories between populations or species, Reise et al. (2006) suggested an empirical scheme of three phases: after one or more introduction events, an establishment phase of low abundance and minor spread may linger on for several generations. It may be followed by exponential expansion phase leading to dominance with strong impacts. The subsequent adjustment phase begins with behavioral and evolutionary adaptations to abiotic and biotic conditions and can be described by three categories (Fig. 1).

If invaders remain rather unaffected by enemies (e.g., due to counter adaptations or newly arriving enemies), the achieved dominance can become permanent (type I). The clam *Mya arenaria* in European estuaries (Strasser 1999) or the Mediterranean mussel *Mytilus galloprovincialis* at rocky shores in South Africa (Branch and Steffani 2004) are good examples. Most invaders, however, undergo strong fluctuations in abundance and population size in the course of the adjustment phase (type II). They may even fail to recover from bust after initial boom (type III), e.g., the comb jelly *Mnemiopsis leidyi* in the Black Sea (Bilio and Niermann 2004) and the polychaete *Marenzelleria viridis* in the Wadden Sea (Essink and Dekker 2002).

We here present a case study of the first 26 years since initial spread of Pacific oysters *Crassostrea gigas* (Thunberg 1793) in the northern Wadden Sea (eastern North Sea). Although *C. gigas* was introduced from a Scottish hatchery since 1971 on a small scale (Drinkwaard 1999a), a feral population only established after 1986 when commercial oyster farming imported annually about 1 million half-grown Pacific oysters from an Irish nursery which in turn received oyster spat from British hatcheries. These oysters are kept in net bags tied to trestles positioned at low tide level, and in winter are usually stored indoors to avoid damage by ice and storms. Public concern about introducing an exotic oyster into the Wadden Sea National Park were overcome by assuring that North Sea waters would be too cold for reproduction. This was contrary to the facts already known at that time (Drinkwaard 1999b; Troost 2010), and a feral population of *C. gigas* established, expanded exponentially and then entered an adjustment phase.

By comparing the first 26 years of feral development with oyster invasions at other coasts, we make an attempt to reveal main factors shaping the dynamics of this population in relation to the relative roles of invasive traits and recipient conditions. For initial phases we can ask what facilitated or inhibited establishment and expansion, while later phases of the invasion can indicate what has stopped the population boom. Answering such questions and providing details from closely monitored invasion processes might provide the necessary data to develop a general model of marine invasions that can be used to implement proper management. In particular, we urge to extend more studies beyond expansion phase to improve understanding and evaluation of biological invasions. The ultimate question will be whether the adjustment phase should be regarded as a general invasion characteristic or more as a specific outcome of life history characteristics and the contingencies of species interactions and environmental variability in the recipient region.

## Methods

**Fig. 2:**
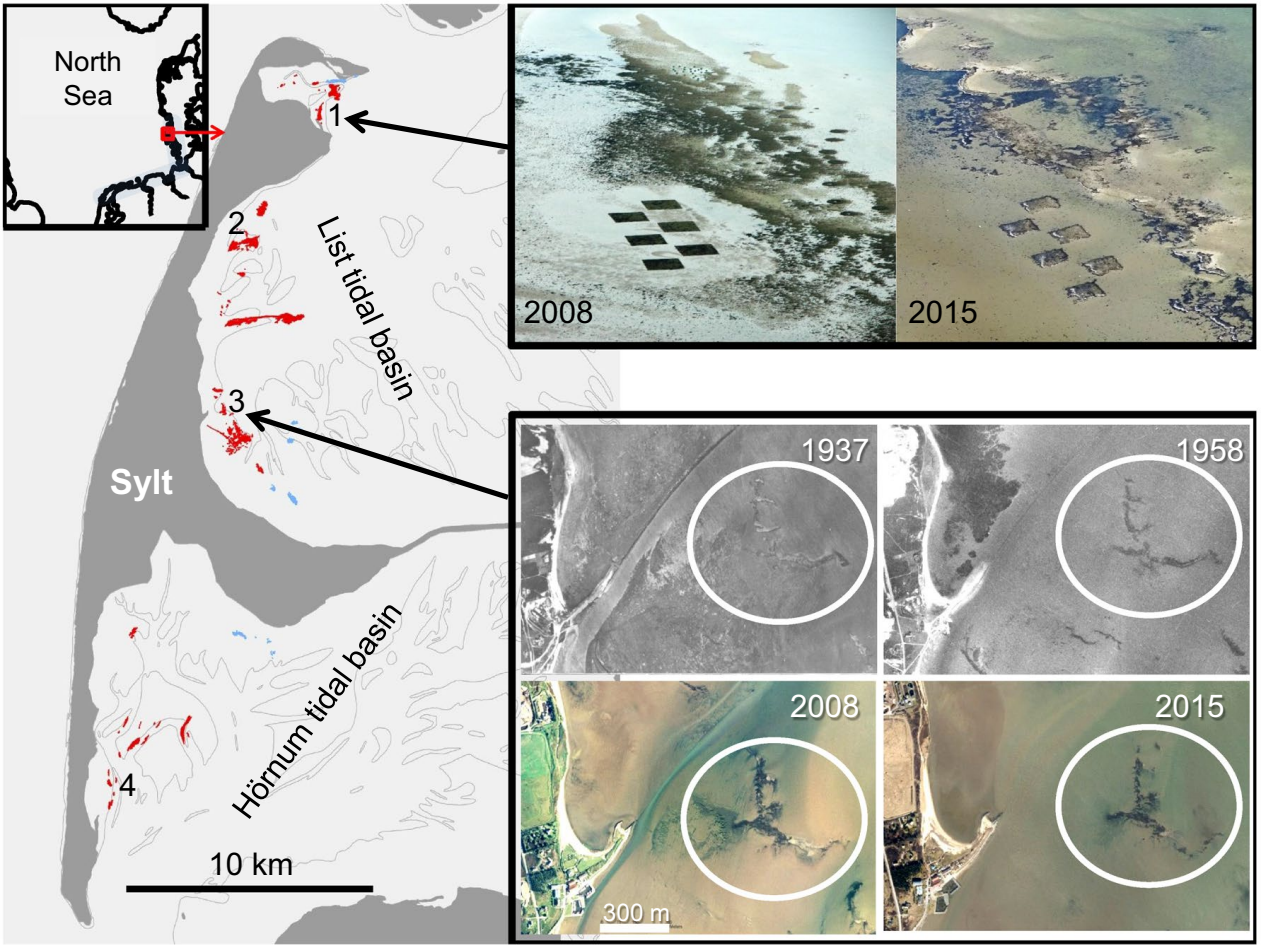
Intertidal beds dominated by oysters (*dark*/*red*) and mussels (*light gray*/*blue*) in terms of total wet weight or cover in 2013 in the tidal basins sheltered by the island of Sylt (for position see *inset* *upper left*), drawn from aerial photographs and by visiting almost all beds on the ground. *Thin line* indicates mean low tide level. Locations mentioned in the text: 1 Königshafen, 2 Oyster farm, 3 Munkmarsch, 4 Puan Klent. *Upper right*: Aerial views of experimental beds in Königshafen on a lugworm flat near a wild bed of mussels and oysters. From 2 months after initiation in 2008 (*left*) to 8 years later the square-cut beds have hardly changed except minor losses in winter. Color intensity varied with algal cover, and on bare sediment with tube caps of the polychaete *Lanice conchilega* abundant in 2008 and wave erosion in 2015. *Lower right* mussel beds near Munkmarsch harbor on aerial photographs taken at low tide long before (*upper row*) and after (*lower row*) Pacific oysters took over. Aerial appearance varies more with picture quality and low tide conditions than with size and shape of the biogenic structure. The annually sampled beds are encircled

In this analysis of the *Crassostrea gigas* invasion since first feral oysters were found near the island of Sylt, we combine (1) the results of an initial assessment of the population in the northern and southern tidal basins at the lee side of Sylt after the first 6 years (see Fig. 2; Reise 1998), with (2) subsequent investigations by Diederich (2005a, b, 2006), Diederich et al. (2005), Nehls et al. (2006), Büttger et al. (2011), Kochmann et al. (2008), Eschweiler (2011) and Moehler et al. (2011) on this regional oyster invasion, and (3) new field data collected on a yearly basis until 2016.

These annual oyster counts were conducted between August and September on mussel beds near Munkmarsch harbor, 5 km south from the oyster farm (Fig. 2). This coherent cluster of mussel beds was visited for counting in 1995, 1999 and regular sampling was done from 2001 to 2016. Total sample area per year varied from 0.24 m² with 6 replicates in 2014 to 28.65 m² with 129 replicates in 2001, depending on oyster density and returning tide (Table 1). Assessments of abundances were done during low tide. Depending on oyster density, quadrats of 0.04 to 1 m² (mostly 0.04 m²) were randomly placed on mussel beds and later on oyster reefs, as oyster density increased. Sampling was stratified with counts confined to areas covered by epibenthic bivalves, whereas bare patches were avoided. At high density, oysters and mussels (*Mytilus edulis*) were collected and washed through a 1-mm mesh sieve before counting. To maximize the number of samples, oyster spat <20 mm were not counted because these were hard to detect when attached to adult oysters.

**Table 1:** Sampling of *Crassostrea gigas* ≥ 20 mm Ø on mussel beds near Munkmarsch harbor (see Fig. 2). Sample size and method (non-destructive visual inspection or picking epibenthos by hand and washed over a 1-mm mesh) was adapted to oyster density. Occasionally the returning tide curtailed the number of replicates

Month-Year	Size of samples (m²)	Number of samples	Total sample size (m²)	Method
08-1993	1.00	485	485	Visual
07-1995	0.25	32	8.0	Visual
09-1999	0.25	126	31.5	Visual^a^
07-2001	0.25	90	22.5	Visual^b^
07-2001	0.16	40	6.4	Visual^b^
09-2002	0.25	40	10	Visual
08-2003	0.04	75	3.0	Visual
09-2004	0.04	70	2.8	Visual
09.2005	0.04	40	1.6	Visual
09-2006	0.04	40	1.6	Visual, 6 sieved
09-2007	0.04	30	1.2	Sieved
09-2008	0.04	14	0.56	Sieved
09-2009	0.04	16	0.64	Sieved
07+09-2010	0.04	34	1.36	Sieved
09-2011	0.04	8	0.32	Visual
09-2012	0.04	10	0.40	Sieved
09-2013	0.04	8	0.32	Sieved
08-2014	0.04	6	0.24	Sieved
09-2015	0.04	7	0.28	Sieved
07 to 09-2016	0.04	8	0.32	Sieved

^a^From Diederich et al. (2005)

^b^These data are combined in Fig. 3 with weighed means and variation

**Fig. 3:**
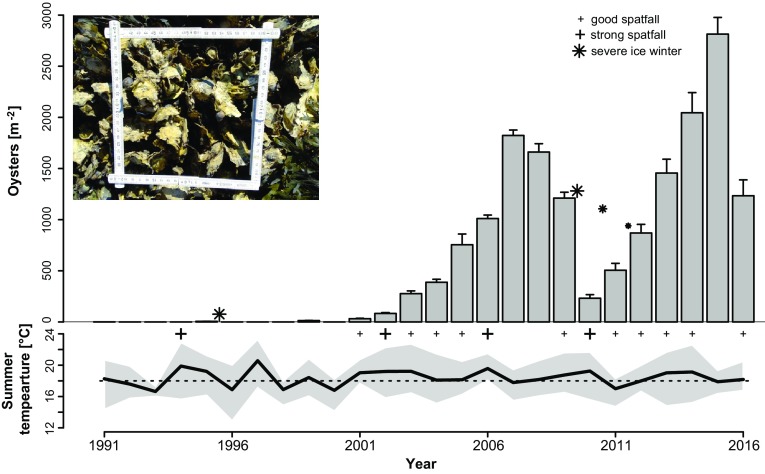
*Upper panel* abundance of *Crassostrea gigas* (≥20 mm) m^−2^ on mussel beds near Munkmarsch (see Fig. 2) from 1993, 1995, 1999 and 2001 to 2016, counted between July and September (see Table 1). *Columns* refer to means and *vertical bars* to standard error. ‘+’ indicates spat of the year was present and ‘**+**’ a particularly abundant spat fall of oysters (<20 mm). Ice winters were very severe in 1995/1996 and 2009/2010 and modest in 2010/2011 and 2011/2012 (size of ✳ indicates relative duration of ice cover). *Inset* shows sample size of 0.04 m². *Lower panel* sea surface temperatures in List tidal basin. *Bold line* shows the mean of measurements in July and August taken during daytime cruises (means were calculated from 13 measurements on average) and the shaded area encompasses the maximal and minimal values measured in the corresponding period. *Dotted horizontal line* indicates assumed 18 °C threshold for spawning (see Diederich et al. 2005)

Although oyster spat was not counted in this survey, qualitative observations on the occurrence of oyster spat were made throughout the tidal basins. Spat distribution was very patchy and categorized as ‘absent’ when in autumn none or only small numbers were found, ‘present’ when variable numbers were observed at >3 sites, and ‘abundant’ when encountered at almost all beds in high numbers. We tested the influence of summer temperature (mean and maximum temperature recorded in July and August during cruises for plankton monitoring carried out by the Wadden Sea Station Sylt) on observing a spatfall event by binomial generalized linear models (GLM) with logit link function. Likelihood ratio tests were used to assess significance of the effects of mean and maximum temperature.

To validate our on site assessment, we compared rapid field counts with careful counting of nine 0.04-m² samples from reefs with a high proportion of small oysters in the laboratory. We found that field counts underestimated oyster abundance by 12% and that of mussels by 6%. Thus, field counts should be taken as proxies of oyster abundances. Shell size was measured in the field to nearest 5-mm interval. Although shapes of oysters could vary considerably, for simplicity only the longest diameter (from umbo to farthest shell edge) was recorded.

To investigate potential habitat expansion during the later stages of the invasion we checked for subtidal colonization. The shallow subtidal zone (to 1 m below mean low tide level) was investigated by wading during exceptional low tides when offshore winds and spring tides coincided. Deeper zones were investigated by dredging (1 m width of metal blade, meshes of 10–20 mm; built after Fig. 3 in Möbius 1877). Depth was measured relative to actual low tide level (http://www.bsh.de/aktdat/bm/Baden&Meer.htm) from nearest tide gauge.

Six experimental oyster plots of 10 × 10 m were set up on a lugworm flat in the eastern part of Königshafen on the landward (sheltered) side of a mixed wild bed with mussels and oysters by collecting scattered clumps of oysters in June/July 2008 (Fig. 2, upper part). Bare sediment was completely covered at experimental plots to measure effects of beds on sediment benthos relative to uncovered areas in between. Here we only compare plot area between 2008 and 2016. Together with aerial surveys on long-term position, size and shape of beds (Büttger et al. 2014 and see Fig. 2, lower part) we use these data to evaluate bed consistency over time. On this basis we assumed a constant area of 0.3 km² of mudflats covered by beds of mussels and oysters (area estimate from Reise and Lackschewitz 1998 and Lackschewitz et al. 2002; neglecting the small Danish sector of the northern tidal basin). This area size was used when extrapolating abundance data obtained at the Munkmarsch beds (see above) to total oyster population size in the Sylt tidal basins.

## Results

### Establishment of Pacific oysters in the northern Wadden Sea

Four years after the farm started production of Pacific oysters on the island of Sylt, breeding and subsequent larval dispersal led to first settlement of feral *Crassostrea gigas* (Fig. 3). In 1991, two young oysters were found 6 to 8 km away from the farm, attached to valves of *Mytilus edulis* and *Mya arenaria*. Diameters were 40 and 48 mm, suggesting settlement in summer 1990 (Reise 1998). Since then, feral oysters have been found every year. Mussel beds offered the necessary firm substrate for larval metamorphosis and happened to occur at positions which provide optimal conditions for feral Pacific oysters to thrive. In 1995, a first survey was conducted in the List and Hörnum tidal basins of Sylt to assess the extent of spread. All 21 intertidal mussel beds of the area were visually inspected and oysters were detected at 17 with an average density of 2–3 oysters m^−2^. Most were 20 to 40 mm in length and had presumably settled in 1994. The largest oyster was 114 mm and probably had settled in 1990. At that time, no Pacific oysters were encountered in the subtidal zone by dredging. Some oysters were found at harbor walls and shore revetments but the majority was confined to mussel beds. The size of the feral population in 1995 was approximately 1 to 2 million oysters on 0.3 km² mud flats completely covered by mussels. Thus, the size of the feral population was similar to the number of *C. gigas* reared in the farm.

Pacific oysters established on mussel beds about ± 0.7 m relative to mean low tide level. Uppermost *C. gigas* were not exceeding 4 h of average low tide exposure during semi-diurnal tides. From 1999 onwards, dredge hauls at 1 to 10 m depth below mean low tide level caught scattered clumps of Pacific oysters. Such stray clumps were also encountered throughout the intertidal zone. Based on this general distribution pattern, we regard the monitored beds near Munkmarsch harbor as sufficiently representative for extrapolating estimated abundances to the entire population size and dynamics of *C. gigas* in the Sylt tidal basins.

### Abundance

Near Munkmarsch harbor a coherent cluster of mussel beds occurred around low tide level covering 2 to 3 ha of mud (Fig. 2). Mussel beds have been known from this site for about 80 years. The establishment phase of *C. gigas* began in the early 1990s (Reise 1998). Abundances remained rather low until 2001 (Fig. 3). From 2001 onwards rapid population growth commenced. Within 7 years abundances of more than 1500 m^−2^ were reached. Although oysters became structurally dominant on former mussel beds since 2004, beds remained remarkably similar in size and shape (Fig. 2), indicating that Pacific oysters required the substratum of mussel beds. Assuming bed area remained constant in the two tidal basins, and densities on Munkmarsch beds were representative, population size in the two tidal basins increased to half a billion (0.5 × 10^9^) in 2007 and about one billion in 2015.

### Recruitment

**Fig. 4:**
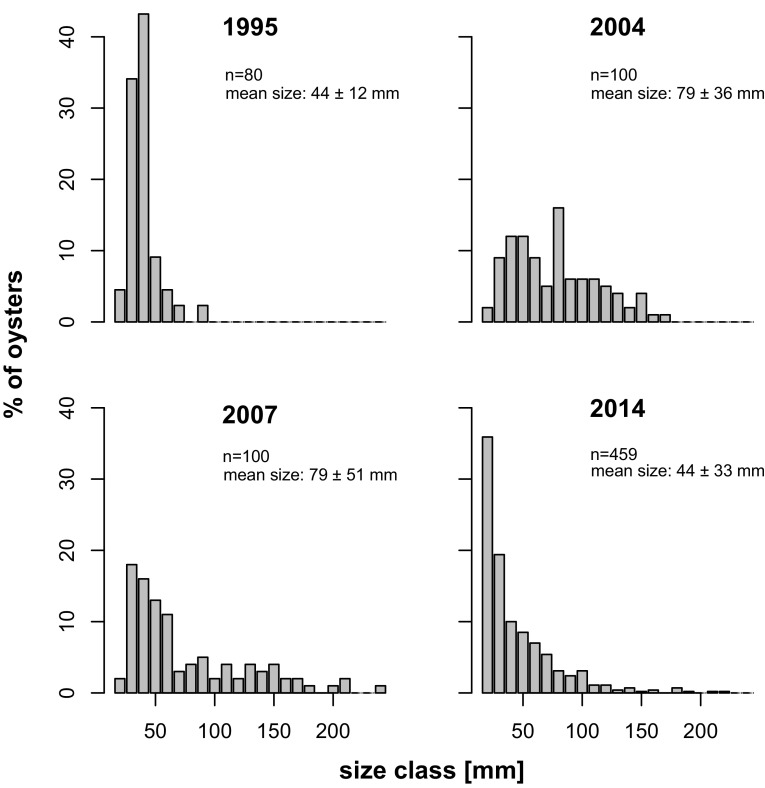
Size-frequency (%) distributions of *C. gigas* near Munkmarsch harbor, not including spat of the year (<20 mm). Often recruits of the previous year dominated numerically, and sizes of >200 mm were present after 2005

Spawning was usually observed in late July, larvae mainly in August and spat <2 mm was found from late August to October. Recruitment did not occur every year. A first strong spat fall apparently happened in 1994. This was not observed directly but can be inferred from abundant young oysters (20 to 70 mm) found in summer 1995 (Fig. 4). Since the oyster farm was established, oyster spat falls occurred in years with July–August mean temperatures exceeding 18 °C (in 6 years from 1987 to 2003; Diederich et al. 2005). In subsequent years, mean summer temperatures were almost always above 18 °C with an increasing but not significant trend from 1987 to 2015 (*y* = 0.037*x* − 56.590; *R* ^2^ = 0.088) leading to particularly strong spat falls in 2002 and 2006, and again in 2010 (Fig. 3). Mean temperature rather than maximum temperature correlated positively with the likelihood of observing spatfalls. This effect was consistently observed for spat falls in general (binomial GLM: mean temperature Deviance (*df* = 1, residual *df* = 25) = 4.629, Likelihood Ratio Test *P* = 0.031) and when only strong spat falls were considered (binomial GLM: mean temperature Deviance (*df* = 1, residual *df* = 25) = 6.860, Likelihood Ratio Test *P* = 0.009). Nevertheless, there was no recruitment observed in 2007 and 2008, after which the population declined. This can be regarded as the beginning of the adjustment phase.

### Winter incursions

Recruitment recurred in 2009, however, after a row of 13 mild winters, sheets of ice mechanically disturbed oyster beds and reefs, and, with freezing conditions at intertidal beds, mortality was high compared to previous winters (Büttger et al. 2011). Mortality affected all age classes but no cohort was wiped out completely. The winter 2009/2010 began with ice floes on December 20th and floating ice sheets were abundant with short interruptions until March 5th. Ice occurred also in the next two winters in February–March. This lasted only over 15 and 14 d, respectively. Mortality was high again but recruitment resumed in the summers in between. Together, the three ice winters caused an intermittent incursion and a reset in population size (Fig. 3), but left ample substrate for new settlement in the form of empty shells.

### Recovery and overall dynamics

Since 2013 winters were mild again. Shell size distribution in 2014 was dominated by juveniles from the two previous years (Fig. 4). Earlier cohorts caused no spikes in distribution from 2014, and very large individuals were few compared to 2007. Population size increased exponentially for a second time, reaching maximum density in 2015 with more than 2500 m^−2^ and exceeding the previous boom of 2007 but at smaller average size. The lack of recruitment in 2015 and very low recruitment in 2016, however, may indicate continued adjustment phase.

In conclusion, the total dynamics over 26 years since first settlement of *C. gigas* on mussel beds near Sylt can be divided into 11 years with rather low and variable abundance (establishment phase), two intervals (7 and 4 years) of exponential population growth (expansion phase), separated by two years without recruitment and three consecutive ice winters that together led to a strong population decline. Recruitment did, however, resume during the phase with ice winters and might have mitigated the losses due to winter mortality. Highest abundance was observed in the last but one year; however, concomitant recruitment failure suggests that adjustment phase rebounds.

### Conservative bed area

Based on observations on the ground and aerial photographs (see Fig. 2 and Büttger et al. 2014), positions and area occupied by beds of mussels and oysters in the tidal basins of Sylt seem to be rather conservative, regardless of whether composed of a mono-dominance of mussels or a co-dominance of mussels with oysters. This observation was supported by an unsuspected side effect of a field experiment with six artificially created plots covered with oysters and mussels (Fig. 2). From 2008 to 2016 these artificial square beds lost merely 11 ± 4% of their original area—in spite of three ice winters in between—and none of the squares expanded. However, due to recruitment since 2010, oyster abundance on the experimental beds tripled from 300 ± 197 to 805 ± 240 m^−2^, *n* = 6, and mussel abundance increased from 741 ± 257 to 930 ± 313 m^−2^, *n* = 6. It also suggests that oyster abundances developed in similar manner on different beds, and overall abundance during the last 26 years can be extrapolated to the approximate population size of feral oysters in the Wadden Sea around Sylt.

### Life span

**Fig. 5:**
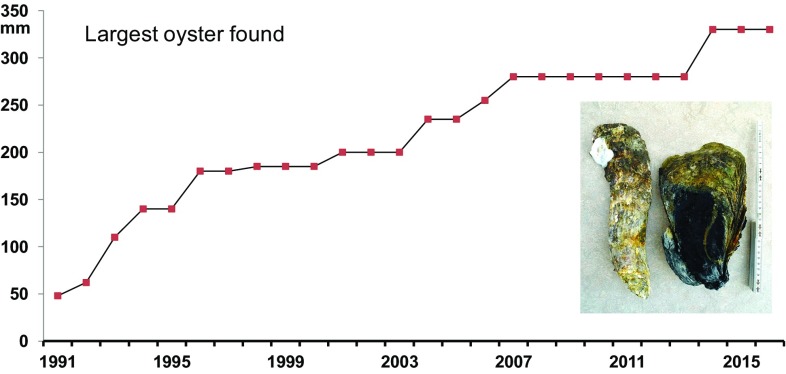
Maximum size (longest diameter in mm) of Pacific oysters encountered in the tidal basins of Sylt from 1991 to 2015 shown cumulatively. Inset shows longest and largest *C. gigas* found (scale 30 cm; see also Table 1)

From an oyster’s perspective, 26 years of invasion may be rather short. The maximum shell size encountered has increased through the entire period, suggesting that some oysters of the early 1990s were still alive in the 2010s (Fig. 5). These old individuals weigh up to 1.5 kg or attain a length of up to 330 mm, and were anchored deep in the sediment (Table 2). Large size, thick shell and partly buried position may contribute to life spans of two decades (or maybe more), because no predators (except man) can be expected to open such big shells and dislocations by currents and waves are hardly possible. Fast upright growth also prevents burial and constitutes an advantage where crowded. However, young oysters growing upon large ones may eventually overtop and then hamper food intake of their basibionts. Accordingly, the largest oysters found usually showed little overgrowth by attached oysters.

**Table 2:** Longest (Puan Klent, July 2014) and largest in terms of weight (Königshafen, March 2016) individual of *Crassostrea gigas* found near Sylt (see also inset Fig. 5)

Oyster parameter	Longest	Largest
Shell		
Max. length (mm)	330	254
Max. width (mm)	95	150
Max. height (mm)	50	55
Below sediment surface (mm)	190	180
Dry weight (3 d at 80 °C; g)	412	1071
Soft body wet weight (cooked; g)	61	75
Dry weight (3 d at 80 °C; g)	10	18
Total wet weight with enclosed water (g)	758	1482

## Discussion

Native oysters *Ostrea edulis* were harvested from wild beds in the northern Wadden Sea until 1925 when the overexploited stock could no longer support commercial use, and the population died out in the 1950s (Neudecker 1990; Seaman and Ruth 1997; Lotze 2005). However, occasionally *O. edulis* were encountered between 1992 and 1995 at low tide level and below. Initially, the oyster farm at Sylt tried to raise this species as well but soon gave up (Thomas Neudecker, perscomm). Presumably, some of these oysters were scattered in the wild but no population became established. The once native oyster beds occurred from low tide level down to about 9 m depth (Möbius 1877). Thus, the feral Pacific oyster population with emerging reefs ±0.7 m around low tide level is overlapping only marginally with the former range of *O. edulis*.

In the twentieth century, the NW-Pacific *Crassostrea gigas* emerged as ‘global champion’ of oyster growers and was introduced to almost all temperate and subtropical coasts, except East-Asia where it came from and NE-America to avoid interference with native *C. virginica* (Chew 1990; Shatkin et al. 1997; Ruesink et al. 2005; Padilla 2010; Beck et al. 2012). *C. gigas* has been successful because of its wide tolerance to environmental conditions, thriving in the intertidal and having high fecundity (Korringa 1976; Quayle 1988). These advantages for oyster farmers were also ideal predispositions of *C. gigas* spreading from sea-based oyster farms into coastal ecosystems. In addition to disclosing the role of adaptive traits, the introductions to so many coasts (i.e., Ruesink et al. 2005; Troost 2010) now offer a unique opportunity to compare invasion trajectories with respect to resistance, accommodation and integration processes in the various recipient ecosystems. In the following, this comparison is conducted stepwise from introduction and establishment, to expansion and adjustment phase (see Fig. 1).

### Introduction and establishment

Since the annual imports of Pacific oysters for farming at Sylt had started, 15 years elapsed before high (>1000 m^−2^) feral densities occurred. Diederich et al. (2005) could show that successful reproduction was bound to a few summers with sufficiently high temperatures ≥18 °C. However, after 4 years larvae dispersed and settled on wild mussel beds. Regular stocking of oysters in the farm circumvented the hurdle of unpredictable temperature conditions for establishment by maintaining propagule pressure. One million densely packed oysters at the farm site may have also facilitated the fertilization rate in the tidal waters. Furthermore, only 50% of the high tide volume of water is exchanged in the tidal basin per semi-diurnal cycle (Gätje and Reise 1998), allowing for retention of planktonic larvae. Finally, abundant mussel beds up- and downstream of the oyster farm offered ample settling sites for oyster larvae at suitable depth in this sedimentary environment.

This combination of favorable prerequisites made the establishment of a feral population around Sylt almost inevitable, and could have been predicted from previous invasions elsewhere in cold waters. The time lag between first introduction and first establishment varied considerably between invaded coastal regions. At the American NW-coast, Pacific oyster introductions commenced in 1912 and first settlement in the wild happened 13 years later (Quayle 1988). In Tasmania, introductions since 1947 gave rise to a feral population 9 years later (Bourne 1979). At the Atlantic coast of southern France, introductions since 1968 were followed by wild settlement already 3 years later (Maurin and LeDantec 1979), while further north at Brittany this took more than 20 years (Lejart and Hily 2011). In the Oosterschelde estuary (SW Netherlands), introductions commenced in 1964 and first settlement happened after 11 years (Smaal et al. 2009). Only at the Swedish coast, where spat was introduced in 1973–1976, establishment took much longer with feral populations observed in 2007 (Wrange et al. 2010). However, it remains questionable whether these feral populations are direct descendents of farmed oysters or rather represent the invasion front from more southern populations unless population genetic studies will be performed.

It seems that first establishment in the wild after the start of sea-based farming was primarily temperature driven. Given the inherent temporal and spatial variability in this parameter, it was like playing ‘ecological roulette’ with oyster establishment bound to happen sooner or later (compare with Carlton and Geller 1993). Once the temperature hurdle was overcome, the almost global distribution of *C. gigas* after a century of introductions suggests that this species with its combination of invasive traits (wide tolerance to physical factors, fast growth, high fecundity, large size and longevity) has encountered little resistance by recipient ecosystems. Nevertheless, establishment phases with low abundances over several years (11 around Sylt) seem to be common, and in several cases have not yet developed further or showed signs of exponential growth. On rocky shores, abundances have generally remained rather low (i.e., Ruesink 2007; Robinson et al. 2005; Krassoi et al. 2008; Kochmann et al. 2013), although localized reefs have developed in parts of southern England (Herbert et al. 2016). This also applies to cold sedimentary coasts in Scandinavia (Wrange et al. 2010; Dolmer et al. 2014; Holm et al. 2015). On British coasts, field trials with *C. gigas* derived from hatcheries in British Columbia, commenced in 1967 and yet high abundances are confined to just a few estuaries (Herbert et al. 2016).

Repeated introductions may have circumvented extinctions after initial establishment. However, also longevity could overcome recruitment failures over many years. At the coast of Argentina, a singular introduction occurred in 1982 because farming was soon abandoned. Nevertheless, an established feral population was observed 10 years later (Escapa et al. 2004), which must be attributed to survivors of the initial introduction. In England, *C. angulata*, which later was recognized as a Taiwanese strain of *C. gigas* (Boudry et al. 1998), was introduced in 1890 and survived in small populations until 1970 (Humphreys et al. 2014; Herbert et al. 2016). Again, this can only be explained by long life spans of a few individuals because no regular spat falls and high abundances were reported.

It can be concluded that ready establishment of *C. gigas* on so many coasts has been made easy by sea-based farming practices with regular introductions of spawning individuals compared to irregular yachts and ships as vectors of introductions. The invasive traits of *C. gigas* made an almost universal establishment inevitable and coastal ecosystems resistant to the invasion of this oyster are few. However, expansion phases leading to exponential growth of feral populations are less ubiquitous.

### Expansion phase

**Fig. 6:**
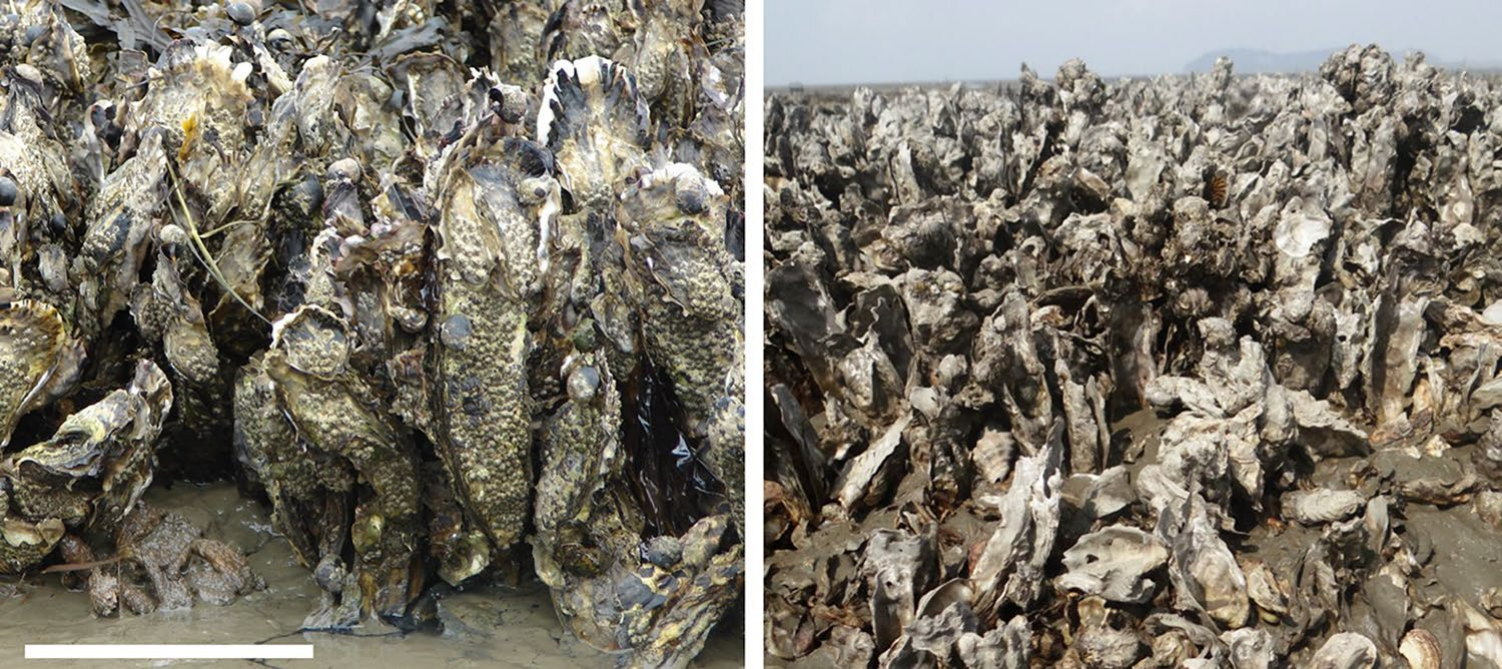
Edge of crowded oyster reef with lateral fusion of valves and attached young oysters on top in the Wadden Sea (Munkmarsch at Sylt) (*left*) and in the Yellow Sea (Daebudo Island, Korea). *Scale bar* 100 mm for both photographs

A transition from less than one hundred oysters per m² to well over 1000 oysters per m² is not common at all. It has not occurred at exposed rocky shores but is a phenomenon of sheltered sedimentary shores where there are larger particles such as gravel, pebbles and shell, rocky outcrops, boulders, harbor walls or biogenic reefs such as mussel beds. Comparisons between coasts are hampered by different methods of quantification in the field, i.e., as percentage of area covered by oysters, as total wet weight or individual numbers per unit area. These are not convertible. At Sylt, abundance of 100 individuals m^−2^ usually amounts to about 25% cover, >400 m^−2^ to 100% cover, and 1000 m^−2^ or more comprise large oysters with shells often partly fused to one another or to empty shells, and with attached younger ones on top, forming a complex reef structure above and below sediment surface, weighing >30 kg m^−2^ (Fig. 6).

The development of such high-density reefs is made possible by larval gregariousness during settlement and by circumventing the larviphagy of adult oysters (Diederich 2006; Tamburri et al. 2007; Troost et al. 2008a, b; Troost 2010). We could show that the proximity of contiguous oysters facilitates larval settlement by conducting an experiment with settlement plates in the centre of artificial rings composed of densely packed mussels, oysters or both species mixed in approximately equal proportion (for further description see Kochmann et al. (2008), and see circular patches at the seaward side of the wild bed in 2008 shown in Fig. 2). Panels surrounded by oysters had significantly more oysters spat than panels surrounded by mussels (unpublished data). Such gregariousness of larval settlement facilitates crowding on oyster reefs and also leads to stable bed areas.

More positive feedbacks may play a role in exponential population growth. The elevated three-dimensional reef structure offers firm substrate for settlement in good feeding positions, and reefs protect individual oysters against dislocation by waves or currents, and probably against predators. Lejart & Hily (2011) mention abundances >1000 m^−2^ for estuarine habitats in Brittany, Cognie et al. (2006) wet weight up to 45 kg m^−2^ south of Brittany, and Laugen et al. (2015) >1000 m^−2^ as upper range for shallow water sites at Swedish fjords. These high abundances may be rather patchy or insular due to limiting attachment for settling oyster larvae. However, in the Wadden Sea and presumably in the nearby Oosterschelde estuary, abundances >1000 m^−2^ on former mussel beds have become a common feature since the 2000s (Markert et al. 2010, 2013; Nehls et al. 2011; Dolmer et al. 2014; Walles et al. 2015 and this study). Apart from harbor walls and low-lying coastal revetments at low-to-moderate wave exposure, such high abundances occur on mussel beds or sites of former mussel beds with abundant empty shells. In the western Dutch Wadden Sea, oysters also covered sites where mussel beds had been overexploited and devastated by a previous fishery (van Stralen et al. 2012; van den Ende et al. 2016). Mussel beds provide the substratum for large and dense Pacific oyster reefs in the Wadden Sea at positions ideal for epibenthic suspension feeders. On a global scale, the Wadden Sea has developed into a hot spot of feral *C. gigas* thanks to its extensive mussels beds.

There seems to be a close match between the ecological niches of oysters and mussels in this coastal region. However, the match is not perfect. Oysters are few and small at the highest parts of mussel beds (own unpubl data; Waser et al. 2016), and there are still regions with mussel beds in the Wadden Sea where oysters have not achieved high abundances (Nehls et al. 2011; van Stralen et al. 2012; van den Ende et al. 2016). Also at the coast of SE-England, in Limfjorden (Denmark) and Kattegat (Sweden), Pacific oysters occur on sediment together with mussels but oyster abundances were one order of magnitude lower than in the Wadden Sea (Groslier et al. 2014; Holm et al. 2015, 2016; Norling et al. 2015; Hollander et al. 2015; Herbert et al. 2016). Insufficient food supply has been suggested as an explanation but the exact mechanisms preventing high oyster densities in these areas are still unclear.

The presence of mussel beds alone is, however, not sufficient to explain the development towards abundances >1000 per m². Temperature is crucial for the global distribution of *C. gigas* (Carrasco and Barón 2010), and for spawning a wide range of 16 to 34 with an optimal range of 20 to 25 °C has been suggested (Shatkin et al. 1997). The role of high summer temperatures for spawning and larval development has been shown to trigger recruitment success in *C. gigas* around Sylt (Diederich et al. 2005). From 2001 onwards, mean July–August water temperatures exceeded 17 °C (except in 2011) and recruitment success became rather regular. Particularly strong spat fall events coincided with peaks of temperature maxima (Fig. 3). Regular recruitment success may be a precondition for crowding to occur with oysters settling upon oysters, generating reefs which become independent of the underlying mussel beds. In conclusion, the transition from establishment phase with low abundances to expansion phase with abundances up to two orders of magnitude higher in the Wadden Sea was aided by common mussel beds on sheltered sediments and high regional summer temperatures (Wiltshire and Manly 2004; van Aken 2008; Witte et al. 2010) that allowed regular recruitment with oysters settling upon oysters.

The rare occurrence of *C. gigas* reefs with abundances >1000 m^−2^ on global scale needs to be investigated further. High-density reefs also occur on mud flats in the native region but individuals tend to remain smaller than in the Wadden Sea (Fig. 6). In the Yellow Sea, these reefs are not grounded on mussel beds but on scattered boulders, often laid out by artisanal fisherman. Boulders may become buried by sedimentation while the oysters grow upwards and subsequent generations settle upon their predecessors. So far it seems that *C. gigas* reefs are confined to sedimentary environments at low tide level with firm substrate for attachment. Further prerequisites are frequent recruitment and probably eutrophic conditions with strong tidal flushing.

### Adjustment phase

After peak density in the feral oyster population around Sylt in 2007, decline commenced with two consecutive summers without recruitment and a series of ice winters starting 2009/2010 when many oysters died. Comparing autumn and spring abundances, Büttger et al. (2011) estimated winter mortality to about 90% with all size classes of *C. gigas* affected. However, most shells remained in place and long-lasting effects on the oyster population were not to be expected. For the same winter, Strand et al. (2012) estimated 66% winter mortality in the adjacent Danish Wadden Sea, while no enhanced mortality was noted further south in the Dutch Wadden Sea (van Stralen et al. 2012). As can be expected, winter mortality at Scandinavian coasts increased with latitude with up to 100% in the course of that unusually severe winter (Strand et al. 2012). Thus, Pacific oyster populations at higher latitudes may experience occasionally dramatic crashes when hit by harsh winter conditions.

However, the long-term ice record for the German North Sea coast shows a general decline in frequency since the mid 1980s (Schmelzer et al. 2016). When oyster abundance was still low and most individuals young, the similarly harsh ice winter of 1995/1996 caused only a mortality of 34% (Reise 1998). Also Büttger et al. (2011) recorded lower winter mortality at sites with lower oyster abundances. They suggested that the high-density population at northern Sylt might have been more vulnerable to sheets of ice moving back and forth with the tides. The following two winters (2010 to 2012) brought some ice cover as well, and recovery of the feral population was delayed. In view of the long period with mild winters (1996 to 2009), the strong winter incursion in the invasion trajectory of Pacific oysters around Sylt should be regarded as a singular event but could have lasting effects by selecting for cold resistant oysters. During the establishment phase, the number of farmed and feral oysters remained in the same order of magnitude and extended gene flow from hatcheries to the feral population could have prevented selection for local adaptations (Moehler et al. 2011). After the expansion phase, however, strong selective pressure such as ice winters or disease on the numerically dominant feral population may facilitate local evolution and influence the extent of future frost and disease-associated mortalities (Sussarellu et al. 2015; Wendling and Wegner 2015; Wendling et al. 2017).

Recruitment failure in 2007, 2008 and 2015 coincided with particularly high abundances of resident oysters. Three years are not yet sufficient to prove this case; however, future research should address density dependence as a driver in the population dynamics of *C. gigas*. According to our own qualitative observations in adjacent areas with lower oyster density, these recruitment failures were a phenomenon confined to the tidal basins of Sylt. Since oyster spat was not only lacking within reefs but also on scattered clumps of oysters, we assume that food shortage or larviphagy is unlikely to explain recruitment failure. More likely could be epidemic diseases arising at high population density, affecting larvae or oysters soon after metamorphosis in particular. Size-frequency distributions (Fig. 4 and see Schmidt et al. 2008; Walles et al. 2015) suggest high mortality in the first year of benthic life. Although shore crabs *Carcinus maenas* and starfish *Asterias rubens* have been observed to prey on small oysters (own unpubl. experiments), predation is unlikely to account for complete recruitment failure in the intertidal zone. Crabs preferred young mussels over young oysters and starfish were almost absent from intertidal flats around Sylt.

However, pathogenic bacteria and viral infections resembling the widespread summer disease syndrome (SDS) are known to affect populations in adjacent regions (Watermann et al. 2008; Thieltges et al. 2013; Wendling et al. 2014; Mortensen et al. 2016) and the recent recruitment failure and population decline (2015/2016) were associated with increased loads of oyster herpes virus (OsHv1, unpublished data). This may suggest that disease can also play an important role for the population dynamics of feral oysters on Sylt. With global warming and continued crowding in a large host population, it is to be expected that *C. gigas* around Sylt will become more frequently exposed to disease agents (see Fey et al. 2015; Sweet and Bateman 2015; Travers et al. 2015). Oyster diseases can affect different life stages but mortalities are usually highest in early life (Le Roux et al. 2016), where also evolutionary responses are most efficient and can further contribute to establishment success (Wendling and Wegner 2015).With the development of a large feral oyster population around Sylt, we predict an adjustment phase with diseases taking a higher toll, causing strong density-dependent fluctuations around a mean density of about 1000 oysters m^−2^ on the reefs, thus resembling the type II trajectory in Fig. 1.

## Summary and outlook

The observed invasion of introduced Pacific oysters in the tidal basins of Sylt during the first 25 years can be seen as a representative model of feral oyster invasions in a global context: Establishment was facilitated by repeated introductions. During the 1990s, recruitment was sporadic because summer temperatures were often too cold for spawning and overall abundance remained low. Since the 2000s warmer summers became normal and recruitment more regular. This triggered the exponential expansion phase. At particularly high oyster densities, recruitment failures were observed, indicating the beginning of an adjustment phase. An incursion of ice winters caused high oyster mortality but had little effect on bed area, and exponential population growth resumed once again, achieving abundances of more than two thousand oysters per m² before recruitment failure recurred.

Once the vagaries of early oyster life have been overcome, high longevity of established individuals constitutes a backbone of population persistence. Large individuals also offer suitable attachment for new generations, initiating a positive feedback which may lead to reef building. Reefs will eventually exceed maximum individual life spans and then further support population persistence. In this way, the Pacific oyster is progressively anchoring itself deeper into the recipient ecosystem, and over time will leave a conspicuous calcareous signal in the geology of the Wadden Sea as well. With the accretion of shell material, reef area may slowly expand but may also raise reefs above 4 h of tidal emergence which seems beyond the physiological range of *C. gigas*.

The invasion trajectory observed at Sylt may be representative for most of the Wadden Sea region and adjacent estuaries. However, strong winter incursions have been shared only with the Scandinavian coasts further north. Abundances well over 1000 m^−2^ are shared with feral populations in sheltered bays at the French Atlantic coast. Scandinavian and British feral populations in suitable habitats may locally achieve similar abundances in the wake of global warming. Pacific oysters on exposed rocky shores seem to remain at relatively low abundances, perhaps caused by resident predators (Ruesink 2007).

For the future, we expect the density on oyster reefs to fluctuate around about 1000 oysters m^−2^ mainly due to density-dependent epidemic diseases in conjunction with continued climatic warming. Large populations as well as transferring oysters for farming purposes could also facilitate the spread of diseases, precluding a phase of population stability. Evolutionary adaptations by disease agents and by the oysters will further contribute to continuous change as will the globalization of coastal biota by ongoing introductions of nonnative species.

As long as sea-based farming of exotic oysters is regarded as acceptable, there was and will be no chance to prevent establishment of feral populations. This is evident from the almost global distribution achieved by *C. gigas*, and could have been expected after the establishment on the NW-coast of America early in the twentieth century. The development towards conspicuous oyster reefs on top of mussel beds in the Wadden Sea World Heritage Site appears to be a high price paid to satisfy a gourmet market. Analyzing the causes and impacts of this globally replicated development can thus provide a lesson for future mariculture practices.
